# Vortex‐Generated Microdroplets Enable Simple, Rapid, and Low‐Volume Measurement of Condensate Volume and Concentration

**DOI:** 10.1002/advs.202511197

**Published:** 2025-08-21

**Authors:** Feipeng Chen, Ho Cheung Shum

**Affiliations:** ^1^ Department of Mechanical Engineering The University of Hong Kong Pokfulam Road Hong Kong Hong Kong SAR 999077 China; ^2^ Advanced Biomedical Instrumentation Centre Hong Kong Science Park Shatin New Territories Hong Kong SAR 999077 China; ^3^ Department of Biomedical Engineering & Department of Chemistry City University of Hong Kong Kowloon Hong Kong 999077 China

**Keywords:** biomolecular condensates, droplet microfluidics, high‐throughput characterization, vortex emulsification

## Abstract

Biomolecular condensates are increasingly recognized for their crucial roles in intracellular organization and the origin of life. Despite their growing importance, efficiently and accurately quantifying condensate volume and concentration remains challenging, hindering the understanding of their properties and functions. Conventional spectroscopy‐based methods for measuring condensate concentration in bulk systems have practical limitations; for example, they require laborious calibration and preparation procedures, large sample volumes, and in vitro analysis. Here, we introduce a microdroplet‐based method that leverages a simple and rapid vortex‐assisted emulsification approach to encapsulate condensates within microdroplets. By determining the microdroplet‐to‐condensate size ratio and partition coefficient in situ, this method enables rapid and calibration‐free quantification of molecular concentrations within condensates. Notably, it reduces processing time by more than tenfold and reduces sample volume and material cost by over two orders of magnitude, while maintaining accuracy comparable to conventional methods. Moreover, this method can precisely quantify subtle variations in condensate volume and concentration under changing environmental conditions, such as salt concentration and stoichiometries. The microdroplet‐based method is anticipated to find broad applicability in fields where precise, efficient, and in situ quantification of condensate volume and concentration is critical, particularly when sample volumes are limited or environmental conditions are dynamic.

## Graphical abstract

This work leverages vortex‐generated microdroplets to encapsulate condensates and quantify their volumes and concentrations. Compared to conventional bulk methods, the technique substantially reduces sample volume, cost, and processing time. Its broad utility sheds light on a wide range of applications, particularly those that require high‐throughput and cost‐effective characterization of condensate properties.

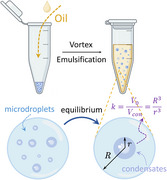

## 1 Introduction

The process of liquid‐liquid phase separation is a fundamental process observed in both biological and synthetic systems, giving rise to biomolecular condensates in cells and complex coacervates in vitro.^[^ ^1^, ^2^, ^3^ ^]^ These condensates form via multivalent interactions among biomolecules and synthetic polymers.^[^ ^3^, ^4^, ^5^ ^]^ Unlike traditional membrane‐bound structures, such as lipid vesicles, condensates lack physical boundaries, allowing their volumes and properties to dynamically adapt to environmental fluctuations in salt concentration, stoichiometry, pH, or temperature.^[^ ^6^, ^7^, ^8^ ^]^ These characteristics underpin their critical roles in spatiotemporal regulation of biological activities^[^ ^9^, ^10^, ^11^, ^12^ ^]^ and the origin of protocells in the prebiotic world.^[^ ^13^, ^14^ ^]^ Despite their significance, the precise and efficient measurement of condensate volume and concentration remains a formidable challenge, significantly limiting our comprehensive understanding of their properties and functions.

**Figure 1:**
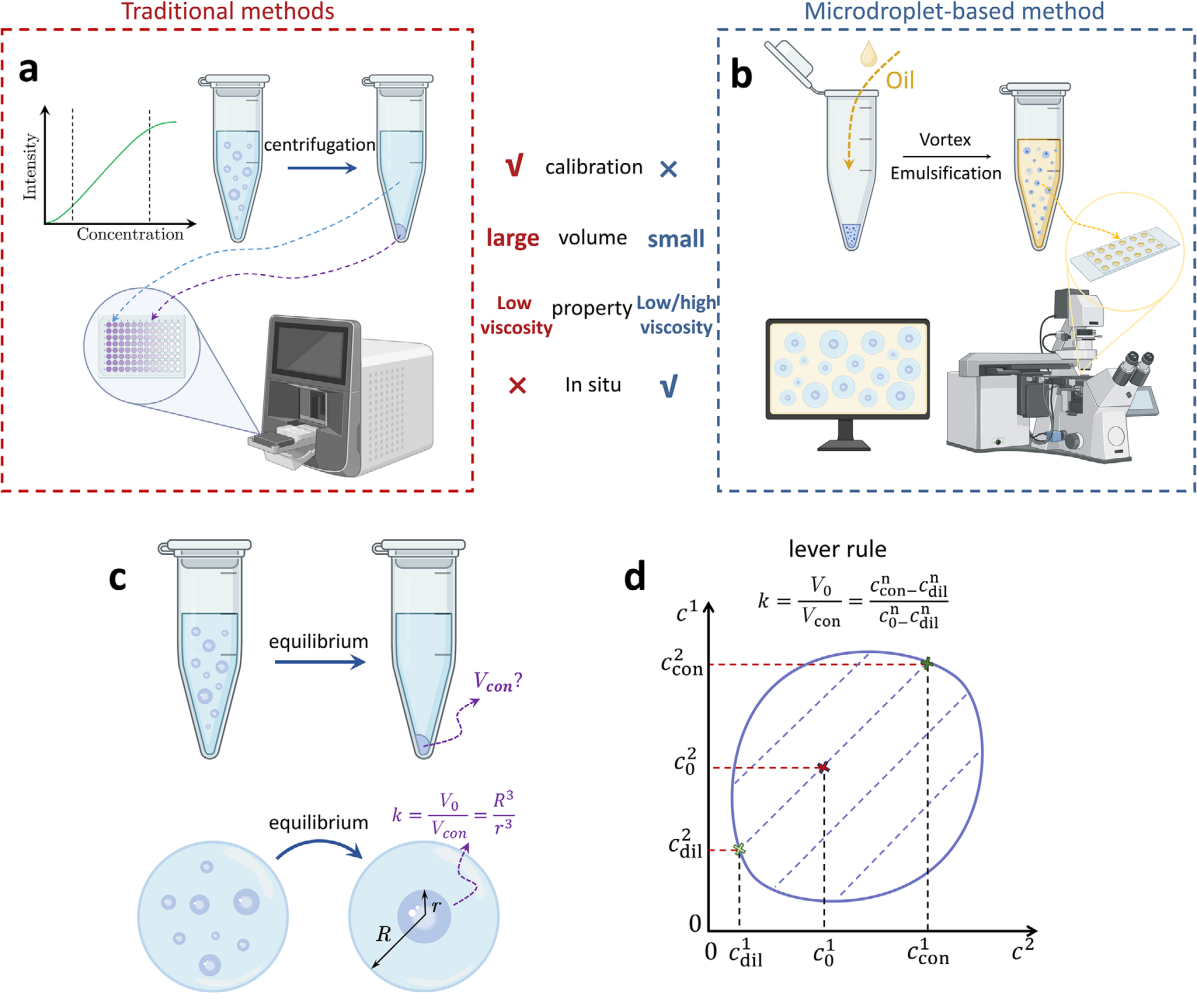
Quantification of condensate concentration by encapsulating condensates into microdroplets. a) Schematic illustration of traditional methods for measuring condensate concentration using Fluorescence microscopy. These methods require establishing a calibration curve. Then, the microtube containing the condensate solution is centrifuged. The bottom condensate phase is then extracted, diluted, and transferred into a 64‐well plate for analysis using a microscopy machine. b) Schematic illustration of our microdroplet‐based method. A small volume of condensate solution is mixed with surfactant‐containing oil in a tube, which is subsequently vortexed for seconds to form stabilized water‐in‐oil microdroplets. These microdroplets are then observed under a confocal microscope for data acquisition. c) Schematic illustration of different geometrical shapes of condensates in a microtube and a microdroplet. d) A phase diagram illustrating a lever rule delineating the relationship between volume ratio and concentration ratio.

Conventional methods for measuring condensate concentration rely primarily on fluorescence or mass spectroscopy techniques (**Figure** **1**a).^[^ ^7^, ^15^, ^16^ ^]^ Although these methods are widely utilized, they have many practical limitations. First, these methods require meticulous calibration to establish a reliable relationship between molecular concentration and detection signal intensity (Figure 1a), which is critical for accurately quantifying molecular concentrations in the dilute and condensate phases.^[^ ^7^, ^17^ ^]^ Subsequent steps involve physically isolating condensates via centrifugation and extracting them from the supernatant.^[^ ^7^, ^18^, ^19^ ^]^ The condensates are then diluted and analyzed using specific spectroscopy techniques, with the final concentration determined by correlating the signal intensity with a pre‐established calibration curve.^[^ ^7^, ^17^, ^18^, ^20^ ^]^ These procedures are time‐consuming, labor‐intensive, and susceptible to errors at multiple stages if not carefully controlled.^[^ ^21^ ^]^

Another crucial limitation lies in the extremely small volume of condensates, which is typically 10^2^ to 10^3^ times smaller than that of the original solution. Therefore, to obtain measurable quantities of condensates, conventional methods typically require large volumes of samples prepared at very high concentrations.^[^ ^7^, ^18^ ^]^ This requirement becomes a hurdle for scarce biological samples, where limited sample availability restricts replicate measurements and compromises the statistical reliability of the data.^[^ ^7^, ^18^ ^]^ These challenges are exacerbated when handling high‐viscous and viscoelastic condensates that are prone to aggregation and solidification at high concentrations.^[^ ^22^, ^23^ ^]^ Extracting these condensates with precise volumes is practically difficult, and condensates may undergo structural changes during the centrifugation and extraction processes.^[^ ^22^, ^24^, ^25^ ^]^ More importantly, existing methods fail to perform in situ analysis of condensates in dynamic conditions, where condensate properties fluctuate in response to environmental changes.^[^ ^6^ ^]^ Collectively, these limitations highlight the urgent need for an efficient and versatile method that simplifies experimental workflows, accommodates diverse condensate types, and facilitates accurate quantification of condensate volume and concentration.

In this study, we encapsulate condensates within microdroplets via a simple and rapid vortex‐assisted emulsification method to measure their volumes and concentrations. By readily quantifying the size ratio of microdroplets to condensates and measuring the partition coefficients, we can determine the condensate volume and the concentration of target molecules within condensates based on the classic lever rule of phase separation. Unlike traditional methods that physically separate and analyze condensates in vitro, our method enables in situ quantification of condensate volume and concentrations and thus preserves their native environments. Furthermore, our method is highly efficient with a more than ten‐fold reduction in processing time and a more than two orders of magnitude decrease in sample volume compared to conventional methods. For example, it requires as little as 4–10 µL of sample, significantly reducing the sample volume and material cost. Hence, our method applies to a wide range of condensates, including those that are very viscous or scarcely available. To demonstrate its utility, we further leverage this method to measure the subtle variations in condensate volume and concentration under varying salt concentrations and stoichiometric ratios. Overall, our microdroplet‐based approach has significantly streamlined experimental procedures and provided an efficient and feasible solution for quantifying condensate volume and concentration, particularly in scenarios where sample volumes are scarce and in situ measurements are essential.

## 2 Measuring Condensate Concentration Based on the Lever Rule

We first demonstrate that condensate concentration can be determined indirectly based on the classic lever rule of phase separation. Upon phase separation, a homogeneous solution demixes into a polymer‐rich condensate phase and a polymer‐deficient dilute phase, with their concentrations denoted as *c*_dil_ and *c*_con_, respectively. These two phases maintain equilibrium due to the balance of the chemical potential of solutes and the osmotic potential of the solvents. In a phase diagram, these two phases are interconnected by tie‐lines, which also go through the initial state, with its concentration denoted as *c*_0_ (Figure 1d). The classic lever rule, derived from the conservation of mass and volume, delineates the ratio of total solution volume *V*_0_ to the condensate volume *V*_con_ as,

(1) $$k=\frac{V_{0}}{V_{\mathrm{con}}}=\frac{c_{\mathrm{con}}-c_{\mathrm{dil}}}{c_{0}-c_{\mathrm{dil}}}\;$$

Under constant environmental conditions (e.g., *c*_0_, temperature, and ionic strength), *c*_con_ and *c*_dil_ remain fixed. Therefore, this lever rule predicts that the volume ratio *k* remains constant, as experimentally validated for condensates both in synthetic systems^[^ ^26^, ^27^, ^28^ ^]^ and in living cells.^[^ ^29^, ^30^ ^]^ Next, we define the partition coefficient of a molecule as the ratio of its concentration in the condensate phase to that in the dilute phase:

(2) $$P=\frac{c_{\mathrm{con}}}{c_{\mathrm{dil}}}\;$$

By combining Equations (1) and (2), we have

(3) $$c_{\mathrm{con}}=\frac{Pk}{P+k-1}\;c_{0}$$

This expression thus enables the determination of condensate concentration *c*_con_ from experimentally accessible parameters, *c*_0_, *k*, and *P*. In conditions where the partition coefficient is much greater than the volume ratio, *P* ≫ *k*, Equation (3) simplifies to *c*_con_ ≈ *kc*_0_. This simplified expression is only applicable in scenarios where condensate volumes are sufficiently large and the partition coefficient of target molecules is large, meaning that all molecules partition into the condensate phase. While the partition coefficient *P* can be measured as the fluorescence intensity ratio of the target molecule between the condensate and dilute phases,^[^ ^8^, ^31^, ^32^ ^]^ precise determination of volume ratio *k* remains as a challenging task.

## 3 Encapsulating Condensates Within Microdroplets to Measure Their Volumes

Volumes of condensates formed in microcentrifuge tubes are inherently challenging to quantify. At equilibrium, condensates merge and sediment to the bottom of the tubes with irregular shapes, complicating precise volumetric analysis (Figure 1a). Previous studies relied on empirical approaches, such as visual inspection or measurement of dilute phase volume, to infer condensate volumes.^[^ ^14^, ^33^ ^]^ However, these approaches are susceptible to error, demand extensive work, and are insufficient for dealing with condensates that are highly viscous or undergo aging transitions (Figure 1a).

We address these challenges by encapsulating condensates within microdroplets (Figure 1b), where the interfacial tension between condensates and the surrounding medium leads to spherical morphologies of condensates. This simple geometry enables a direct calculation of the condensate volume as *V*_con_ =  4π*r* ^3^/3 by measuring the condensate radius *r*, eliminating the need for physical extraction of condensates. As a result, the volume ratio *k* can be readily determined as the cube of the radius ratio between the microdroplet and condensate as,

(4) $$k=\frac{V_{0}}{V_{\mathrm{con}}}=\frac{R^{3}}{r^{3}}\;$$

The idea of encapsulating condensates within microdroplets facilitates the accurate quantification of condensate volume and can be further applied to measure condensate concentrations based on Equation (3).

## 4 Measuring Condensate Concentration Using a Vortex‐Assisted Emulsification

**Figure 2:**
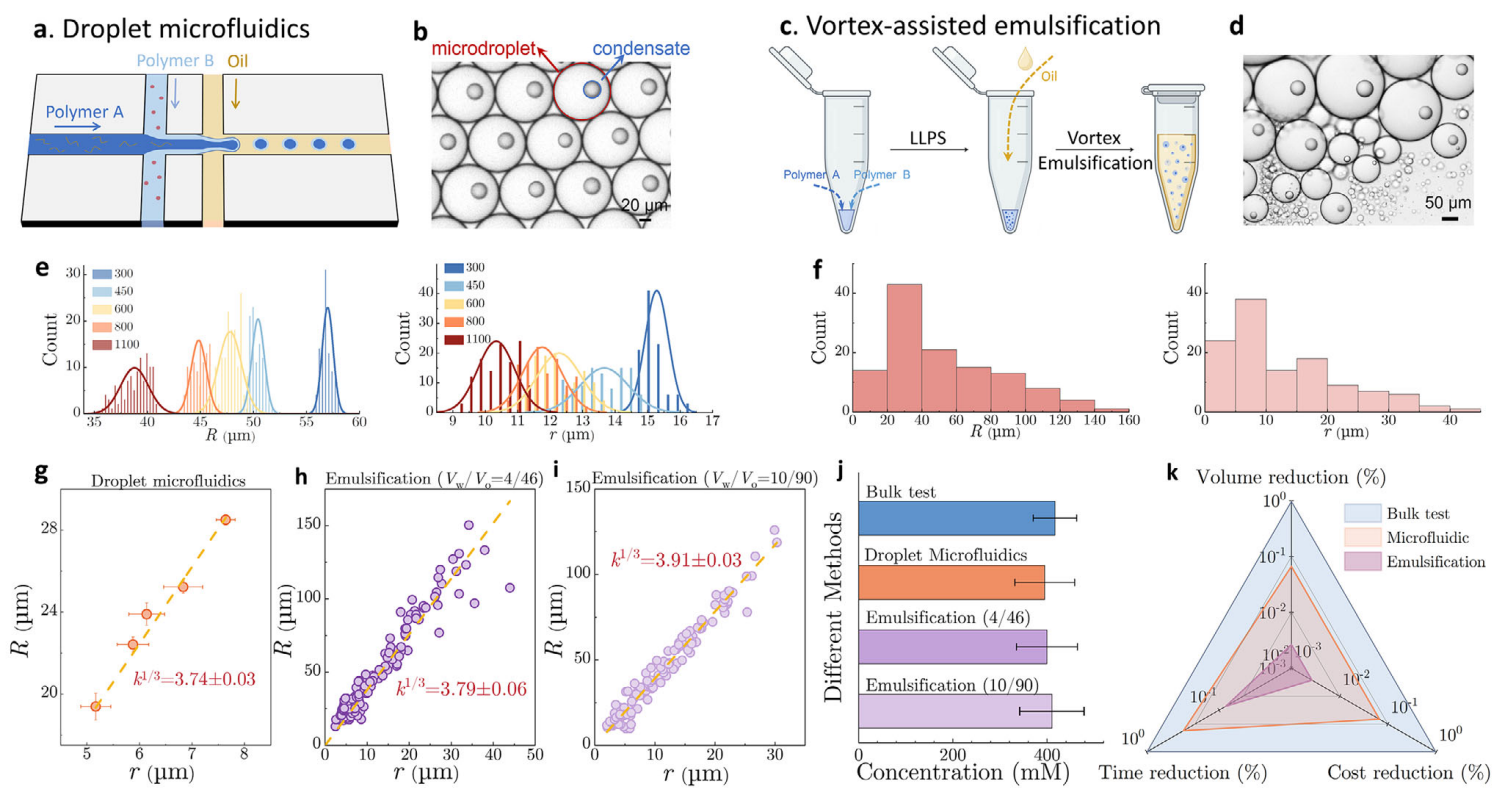
Measurement of condensate volume and concentration in microdroplets via vortex‐assisted emulsification. a) Schematic illustration of a two‐junction microfluidic chip with two inner aqueous phases and one outer oil phase. b) Bright‐field image of condensate‐containing microdroplets generated by droplet microfluidics. c) Schematic illustration of the vortex‐assisted emulsification method to generate microdroplets. d) Bright‐field image of inhomogeneous condensate‐containing microdroplets produced via vortex‐assisted emulsification. Size distribution of condensates (*r*) and microdroplets (*R*) generated using e) microfluidics and f) vortex‐assisted emulsification. Plots of microdroplet radius (*R*) as a function of condensate radius (*r*) for samples prepared using g) microfluidics and h,i) vortex‐assisted emulsification at water/oil volume ratios of 4/46 and 10/90, respectively. j) Values of condensate concentration measured using traditional fluorescence spectroscopy, microfluidics, and vortex‐based emulsification methods (n ≥ 3 independent samples). Data are presented as mean ± SD (n ≥ 3). k) Comparison of reduction ratio (%) in sample volume, processing time, and material costs using different methods for quantifying condensate concentrations. All condensates are comprised of PLL at 20 mm and ATP at 60 mm.

To prove this concept, we utilize model condensates consisting of poly‐L‐lysine (PLL) and adenosine triphosphate (ATP). Encapsulation of condensates within microdroplets is often achieved using droplet microfluidics, which offers precise control over the size of the microdroplets.^[^ ^26^ ^]^ Therefore, we have designed a two‐junction microfluidic chip (**Figure** **2**a). In this setup, laminar co‐flows of PLL and ATP aqueous solutions converge at the first junction, followed by pinch‐off into monodisperse water‐in‐oil (W/O) microdroplets by the outer oil phase. These microdroplets are stabilized by biocompatible surfactants dispersed in the oil phase to prevent them from coalescing. Upon generation, small condensates form and diffuse within the microdroplets (Figure S1, Supporting Information). Over time, these small condensates coarsen and eventually form a single, spherical condensate within each microdroplet, leading to monodisperse pairs of condensates and microdroplets (Figure 2b).

We next systematically vary the oil‐phase flow rate while maintaining constant aqueous phase flow rates and solute concentrations. Increased oil flow rates enhance shear forces at the aqueous‐oil interface, yielding smaller microdroplets (Figure 2e). Interestingly, a similar reduction is observed for the size of condensates within microdroplets (Figure 2e). By correlating their sizes at different oil‐phase flow rates, we find that condensate radius scales linearly with microdroplet radius, yielding a constant scaling ratio *k* ^1/3^ =  3.74 ± 0.03 (Figure 2g).

While droplet microfluidics enables precise control over microdroplet sizes, its operation requires specialized equipment and considerable effort in the design and preparation of the microfluidic devices (see Experimental Section for details). To simplify the condensate encapsulation process and make it more accessible, we ask whether a vortex‐assisted emulsification approach could yield comparable results (Figure 2c). To validate this, 2 µL each of PLL and 2 µL of ATP solutions are homogenized within a microtube using a pipette. Next, 46 µL of oil solution is added into the microtube containing PLL/ATP mixture, followed by vortexing the entire solution for 30 s. At equilibrium, this method generates pairs of condensates and microdroplets (Figure 2d). However, the condensates and microdroplets remain polydisperse, in contrast to the monodisperse size distribution of those prepared by droplet microfluidics (Figure 2f).

Surprisingly, despite sacrificing size uniformity, this method preserves a linear scaling relationship between microdroplet radius *R* and condensate radius *r*, yielding *k* ^1/3^ =  3.79 ± 0.06, which is nearly identical to the ratio obtained using droplet microfluidics. (Figure 2h). To further validate this finding, we test a variety of volume combinations of aqueous and oil solutions. Remarkably, we consistently observe that, regardless of W/O volume ratios, the scaling ratios remain comparable across all these experiments (Figure 2i; Figure S2, Supporting Information). These results thus demonstrate that vortex‐assisted emulsification can reliably generate condensate‐microdroplet pairs with a robust size scaling relationship, providing a much simpler yet effective way compared to droplet microfluidics.

Next, we determine the concentration of PLL within PLL/ATP condensates based on Equation (3). The partition coefficient of PLL is measured to be ≈22.5±5.1 using fluorescein isothiocyanate (FITC)‐labeled PLL and confocal microscopy. As a result, the PLL concentration is determined to be 394.9±63.3, 399.5±64.8, and 410.0±68.2 mm based on the volume ratios obtained by droplet microfluidics and vortex‐assisted emulsification at two W/O volume ratios, 4/46 and 10/90, respectively (Figure 2j). To validate the accuracy of our microdroplet‐based method, we also measure the PLL concentration in the condensate phase using the conventional fluorescence spectroscopy technique (see Experimental Section for details). The PLL concentration determined by conventional methods is found to be ≈416.4±46.10, which is very close to values obtained using our microdroplet‐based method (Figure 2j), validating the accuracy of our microdroplet‐based method.

Critically, our method reduces the required sample volume and material cost by more than two orders of magnitude while simultaneously decreasing processing time by over tenfold (Figure 2k). For example, conventional bulk measurements require at least 3000 µL of sample solutions for three independent tests. In contrast, it only takes 4–10 uL of sample solution using vortex‐assisted emulsification, or ≈100 uL when using droplet microfluidics (Figure S3, Supporting Information). Remarkably, each microdroplet, considered as an independent test, only contains 20–50 pL sample solution, representing approximately eight orders of magnitude reduction in sample volume compared to bulk measurement. In addition, alternative droplet generation techniques (e.g., membrane emulsification) also typically require large sample volumes (>100 µL) and demand substantial time and material costs for the preparation of emulsion droplets. In contrast, our microdroplet‐based method not only maintains comparable detection precision but significantly improves detection efficiency by reducing the processing time and sample cost, making it a highly powerful method to measure the molecular concentrations within condensates.

## 5 Simultaneous Concentration Measurement of Multiple Scaffold and Client Molecules in Condensates

**Figure 3:**
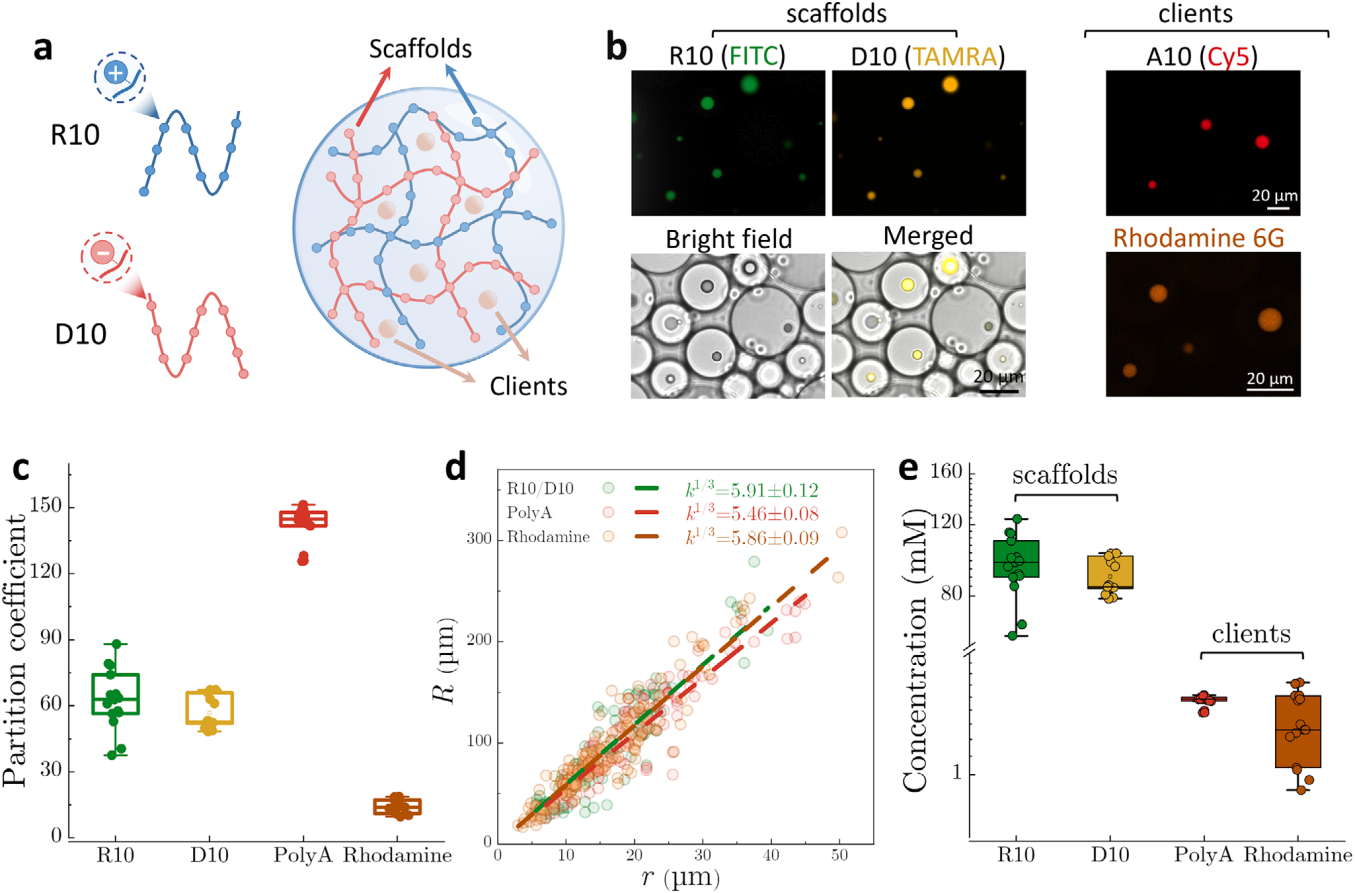
Simultaneous concentration measurement of multiple scaffold and client molecules within the same condensates. a) Schematic illustration of condensates formed by R10 and D10 scaffolds, along with partitioned client molecules. b) Fluorescence and bright‐field images showing the partitioning of scaffold molecules (R10 labelled with FITC and D10 labelled with TAMRA) and client molecules (Cy5‐labelled A10, 0.1 mm, and Rhodamine 6G, 0.02 mm) within condensates formed by R10 and D10 at equal monomeric concentrations of 2 mm. c) Partition coefficients of different molecules inside the R10/D10 condensates. d) Plots of microdroplet radius (*R*) as a function of condensate radius (*r*) for samples consisting of R10/D10 condensates and different client molecules, including polyA and Rhodamine 6G. The size ratios are as follows: 5.91±0.12 for R10/D10, 5.46±0.08 for R10/D10/polyA, and 5.86±0.09 for R10/D10/Rhodamine condensates. These results showing comparable size scaling ratios validate the reliability of the microdroplet‐based method. e) Calculated concentrations of different molecules, including R10, D10, polyA, and Rhodamine 6G, within the R10/D10 condensates.

Conventional methods for quantifying the concentration of multiple molecules within condensates remain labor‐intensive and technically challenging, requiring individual calibration for each target molecule and cumbersome preparation protocols. In contrast, our microdroplet‐based method enables efficient, simultaneous quantification of both scaffold and client molecule concentrations within the same condensate system (**Figure** **3**a). To demonstrate this, we form a model condensate using decapeptides of arginine (R10) and aspartic acid (D10) as complementary scaffold molecules interacting through electrostatic interactions (Figure 3a). Condensates are prepared at 1:1 stoichiometry ratio of positively and negatively charged monomers unless otherwise mentioned. Polyadenylic acid (PolyA, 20mer, cyanine5‐labelled) and rhodamine 6G are incorporated as client molecules.

We observe that R10/D10 condensates are very viscoelastic and exhibit strong adhesion to the bottom wall of microtubes upon centrifugation (Figure S4, Supporting Information). This property makes it difficult to extract the condensate and measure the concentration of target molecules. Instead, these condensates containing locally concentrated R10 and D10 appear as spherical droplets within microdroplets, formed using the vortex‐assisted emulsification approach (Figure 3b). In addition, polyA and rhodamine 6G are also enriched within the condensates, as shown by their enhanced fluorescence intensity in the condensate phase compared to the surrounding dilute phase (Figure 3b). Quantitative assessment of partition coefficients for these scaffold and client molecules reveals comparable values for R10 (62.6±13.2) and D10 (56.9±7.1) (Figure 3c). The negatively charged polyA displays a higher partition coefficient of 142.1±8.2, likely driven by strong electrostatic interactions with the positively charged D10 scaffold molecules (Figure 3c). Conversely, rhodamine 6G exhibits a modest partition coefficient of 14.4±2.9 (Figure 3c), suggesting a less hydrophobic chemical environment within the R10/D10 condensate, consistent with weak hydrophobic effects in condensate systems dominated by electrostatic interactions.^[^ ^34^ ^]^

We then observe that the microdroplet‐to‐condensate radius ratios remain comparable across different condensate systems, comprising R10/D10 (*k* ^1/3^ = 5.91±0.12), R10/D10/polyA (*k* ^1/3^ = 5.46±0.08), and R10/D10/rhodamine (*k* ^1/3^ = 5.86±0.09), respectively. Notably, despite the viscous nature of R10/D10 condensates, our method enables robust determination of size scaling, with data showing minimal deviations from the fitted linear curves. These results suggest that these client molecules, when present at low concentrations, exert negligible influence on the phase separation behavior of R10/D10 condensates. Using the measured radius ratios and partition coefficients, we calculate the concentrations of scaffold and client molecules within the R10/D10 condensate. R10 and D10 have similar concentrations inside the condensate, 95.7±15.8 mM and 89.3±8.7 mM, reflecting ≈50‐fold enrichment relative to the initial concentration *c*_0_. In addition, the concentrations of polyA and rhodamine 6G are ≈1.52±0.05 mM and 1.33±0.24 mm, representing ≈76‐fold and ≈13‐fold enrichment, respectively. Therefore, our method eliminates the need for molecule‐specific calibration, enabling simultaneous quantification of multiple targets within the same condensates. This feature positions our approach as a scalable and high‐throughput platform for screening biomolecular condensates in various applications, such as drug screening.

## 6 Variation of Condensate Volume and Concentration at Different Salt Concentrations

**Figure 4:**
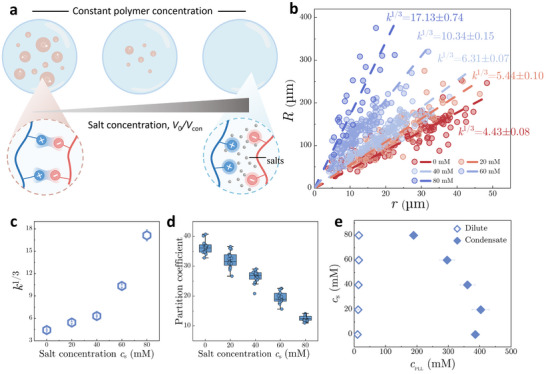
Variations in condensate volume and concentration at varying salt concentrations. a) Schematic illustration showing the reducing condensate volume with increasing salt concentration due to the screened electrostatic interactions at high ionic strength. b) Plots of microdroplet radius (*R*) as a function of condensate radius (*r*) for condensates formed by PLL (15 mM) and ATP (45 mm) at various salt concentrations. c) Variation of the microdroplet‐to‐condensate size ratio *k* ^1/3^ as a function of salt concentration *c_s_*. d) Changes in the partition coefficient *P* of PLL within PLL/ATP condensates with increasing *c_s_*. e) Calculated concentrations of PLL in the dilute phase (hollow diamonds) and condensate phase (solid diamonds) at various salt concentrations. Data are presented as mean ± SD (n ≥ 3).

Biomolecular condensates are inherently responsive to physicochemical cues, and understanding their adaptive behavior is essential for deciphering how cells regulate compartmentalization under stress and deregulation^[^ ^10^, ^26^ ^]^ and how protocells can survive and proliferate in the prebiotic world.^[^ ^14^, ^35^ ^]^ However, quantifying the sensitivity of condensate properties to dynamic environmental changes remains challenging and poorly controlled using conventional methods in bulk systems. To address this, we leverage our microdroplet‐based approach to measure subtle, salt‐dependent variations in condensate volume and concentration (**Figure** **4**a). Salts are particularly effective modulators of phase separation, as they screen electrostatic interactions and thus affect the process. By maintaining a fixed monomer charge ratio of ATP to PLL at 3, we systematically probe how ionic strength influences condensate volume and concentration.

The microdroplet‐to‐condensate size ratio increases sharply with increasing salt concentration *c_s_* (Figure 4b), from *k* ^1/3^ =  4.43 ± 0.08 at *c_s_* =  0 mm to *k* ^1/3^ =  17.13 ± 0.7 at *c_s_* =  80 mm (Figure 4c), indicating a continuous dissolution of condensates. Concurrently, fluorescence measurements reveal that the partition coefficient of PLL decreases with increasing *c_s_* (Figure 4d). These findings are consistent with salt‐induced screening of electrostatic interactions and destabilization of condensates. Calculation of PLL concentrations in both condensate and dilute phases yields a typical phase diagram that narrows as *c_s_* increases (Figure 4e). Intriguingly, the condensate‐phase PLL concentration exhibits a nonmonotonic “looping‐in” trajectory, initially increasing with low *c_s_* before declining at higher *c_s_*. This behavior potentially arises from the nonstoichiometric mixing between PLL and ATP, where incremental salt ions first neutralize excess charged residues and thus promote phase separation, before completely dissolving condensates.^[^ ^36^ ^]^ Overall, these results underscore our method's capacity to monitor subtle and salt‐dependent shifts in condensate properties. Beyond ionic strength, this platform is readily adaptable to interrogate diverse environmental variables, such as temperature, pH, and osmotic pressure.

## 7 Stoichiometry‐Dependent Variations in Condensate Volume and Concentration

In addition to environmental stimuli, the stoichiometric ratio of oppositely charged polymers critically influences the driving forces and material properties of condensates.^[^ ^20^, ^26^ ^]^ However, bulk measurements often fail to resolve stoichiometric effects, particularly at highly asymmetric stoichiometries, where phase separation is significantly suppressed and condensate volumes become very small. Therefore, extracting and measuring condensates using conventional methods is challenging and highly cost.

**Figure 5:**
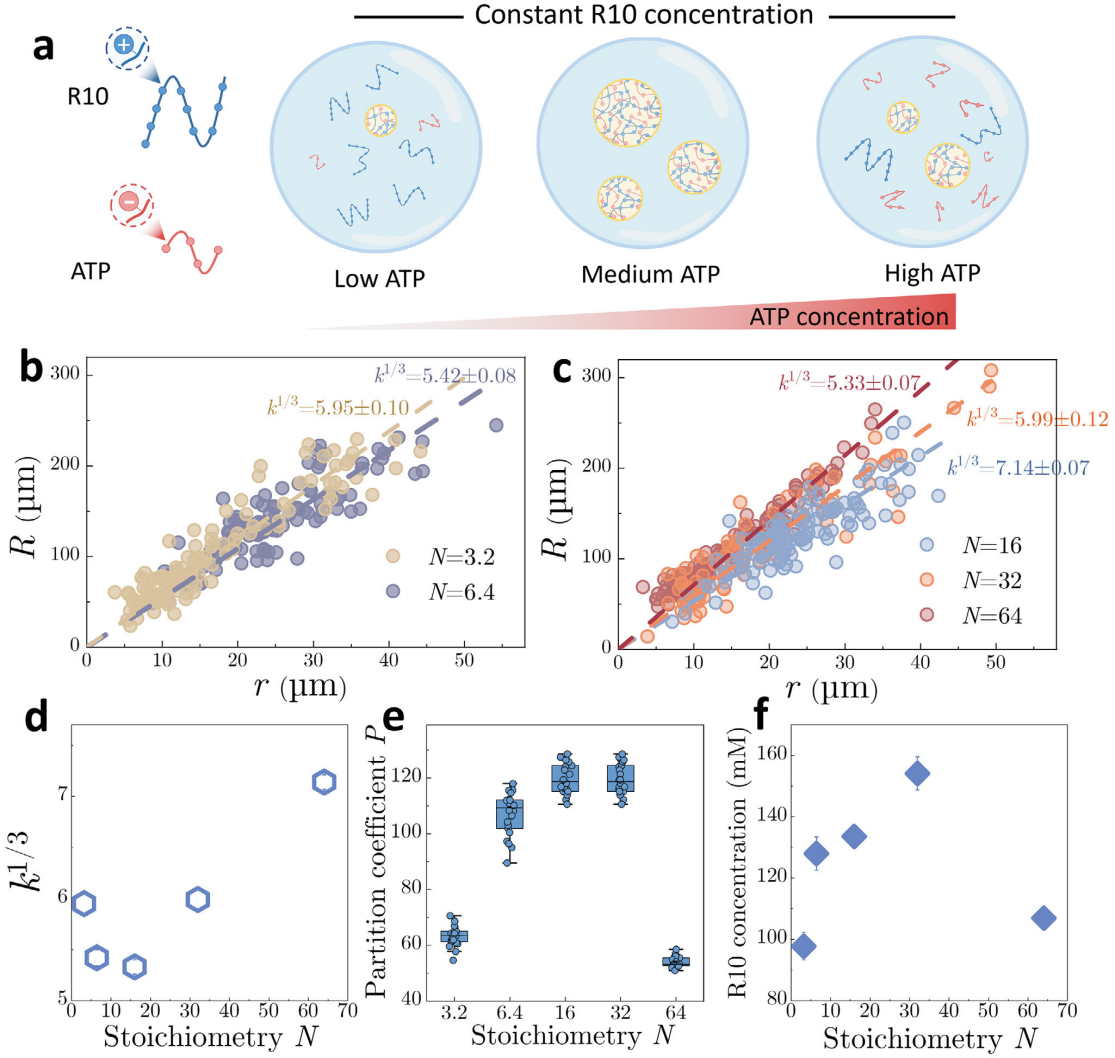
Variations in condensate volume and concentration at varying stoichiometries. a) Schematic illustration showing the changes in condensate volume and concentration affected by the stoichiometry *N* between R10 and ATP. For all condensates, R10 concentration is maintained constant at 2 mm, while ATP concentration is gradually increased. b,c) Plots of microdroplet radius (*R*) as a function of condensate radius (*r*) for R10/ATP condensates formed at different stoichiometries. d) Variation of the microdroplet‐to‐condensate size ratio *k* ^1/3^ as a function of stoichiometry *N*. e) Variation of the partition coefficient *P* as a function of *N*. f) Calculated R10 concentration inside R10/ATP condensates formed at varying stoichiometries. Data are presented as mean ± SD (n ≥ 5).

To solve this problem, we employed our microdroplet‐based method to systematically investigate how condensate volume and concentration vary with stoichiometry in a model condensate system comprising positively charged R10 decapeptide and negatively charged ATP (**Figure** **5**a). Maintaining the R10 concentration constant at 2 mM, we incrementally increase ATP concentration, defining the stoichiometry *N* as the ratio of ATP anionic residues to R10 cationic residues. We hypothesize that maximal condensate volume and R10 enrichment would occur at intermediate *N*, where electrostatic interactions between residues of R10 and ATP are optimally saturated (Figure 5a). Conversely, extreme stoichiometries (low or high *N*) would suppress phase separation (Figure 5a).

As a validation, condensates assembled at *N* from 3.2 to 64 are encapsulated via the vortex‐assisted emulsification method. At *N*<3.2, we observe that condensates remain highly stable without coarsening into a single large droplet over 2 days (Figure S5, Supporting Information), probably due to the high surface charges, which prevent condensates from merging.^[^ ^37^, ^38^ ^]^ By further correlating droplet and condensate radii (Figure 5b,c), microdroplet‐to‐condensate size ratios *k* ^1/3^ shows a nonmonotonic relationship with *N*, initially decreasing to a minimum at *N*  =  16 and then rising at higher *N* (Figure 5d).

Similarly, the partition coefficient *P* of R10 within condensates exhibits a re‐entrant behavior, first increasing and then decreasing over *N* (Figure 5e). Calculation of R10 concentration within the condensates mirrors this trend, with maximal concentration (∼ 154 mM) around *N*  =  16 (Figure 5f). These results confirm that phase separation is enhanced by an optimal stoichiometric balance. Notably, this stoichiometry‐dependent modulation has also been observed in condensates formed by intrinsically disordered proteins with different RNAs, which might serve as a critical feedback mechanism for transcription regulation.^[^ ^8^ ^]^ Overall, these results underscore our method's ability to resolve subtle stoichiometry‐induced changes in condensate volumes and concentrations.

## 8 Conclusion

By encapsulating condensates within microdroplets via vortex‐assisted emulsification, we have developed a versatile method that enables efficient and accurate quantification of condensate volumes and concentrations. Compared to conventional spectroscopy‐based methods, which require laborious calibration and preparation steps, our approach eliminates these constraints while accommodating condensates with diverse material properties, including very viscoelastic condensates. Critically, our method reduces sample volumes to 4–10 µL per experiment, which represents approximately two orders of magnitude reduction compared to bulk assays. This advantage dramatically lowers costs and enables studies on scarce biomolecules.

The ability to observe changes in condensate properties in dynamic environments is pivotal for understanding their roles in cellular stress adaptation, transcription regulation, and pathological aggregation.^[^ ^5^, ^10^, ^39^ ^]^ Traditional bulk measurements, however, disrupt native conditions of condensates during physical extraction. Our microdroplet platform circumvents this by enabling in situ measurements of condensate volumes and concentrations, as exemplified by monitoring salt‐induced destabilization of PLL/ATP condensates and stoichiometry‐dependent phase separation of R10/ATP condensates. These findings align with observations in transcriptional condensates, where ionic strength and RNA‐protein stoichiometries dynamically regulate their phase behaviors.^[^ ^8^ ^]^ Overall, we anticipate that our method will become a widely adopted tool for studying both the equilibrium and dynamic behaviors of condensates. Its broad utility further warrants the exploration of potential applications in future research, such as high‐throughput screening of anti‐condensate therapeutics.

## 9 Experimental Section

### Materials

Poly‐L‐lysine hydrobromide (PLL, Mw≈30000–70000), Fluorescein isothiocyanate (FITC) labeled Poly‐L‐lysine (FITC‐PLL, Mw≈30000–70000), Adenosine 5′‐triphosphate (ATP), Rhodmaine 6G were purchased from Sigma‐Aldrich. Poly(l‐arginine hydrochloride) (degree of polymerization *n* = 10)(R10) and poly(l‐aspartic acid sodium salt) (degree of polymerization n = 10) (D10) were purchased from Alamanda Polymers, USA. FITC‐labelled R10 was purchased from GenScript, Hong Kong. TAMRA‐labelled D10 was purchased from Biomatik, Canada. PSolyadenylic acid (PolyA, 20mer, cyanine5‐labeled) was purchased from Integrated DNA Technologies, USA. All synthesized peptides and nucleotides have a purity higher than 95%. HFE 7500 oil was purchased from 3 m Science, and fluorosurfactant was purchased from RAN Biotechnologies, USA. Sodium Chloride (5 m solution) and UltraPure DNase/RNase‐Free Distilled Water were purchased from ThermoFisher Scientific. All chemicals were used as received without further purification.

### Condensate Preparation

Solutions containing peptides, nucleotides, and small molecules were first prepared at relatively high concentrations by dissolving them using ultrapure distilled water. Fluorescence‐labeled materials were mixed with corresponding unlabeled ones at a molar ratio of 1:24. Solutions were added and mixed to form condensates according to the required solute concentrations in different experiments. In bulk microtube experiments, the sample solutions were vortexed and centrifuged at 4000 g for 10 min to separate condensates from the supernatant. In microfluidic experiments, the microdroplets were incubated at room temperature for approximately one day after droplet generation.

### Image Analysis

Bright‐field and fluorescence images were acquired using a Carl Zeiss LSM 980 confocal laser scanning microscope. For droplet microfluidics experiments, images were processed with Image J using image binarization to delineate boundaries of microdroplets and coacervates. The “Analyze Particles” function in Image J was then applied to the binarized images to measure the areas of microdroplets and condensate. The radii of microdroplets and condensates were subsequently calculated based on the measured areas. For vortex‐assisted emulsification experiments, the acquired images were analyzed using a customized MATLAB script. Spherical circles were drawn to overlap with the targeted microdroplets and condensates to determine their radii. The partition coefficient was measured as the ratio of fluorescent intensity between the condensate and dilute phases using the ZEN software.

### Concentration Characterization

The condensate solution in a microcentrifuge tube was centrifuged at 4000 g for 10 min to separate condensate from the supernatant. 2 µL condensate phase was then extracted and diluted 50 times using 1 m NaCI solution. The absorption value of 100 µL diluted condensate phase was measured using a microplate reader (Molecular Devices) at the characteristic wavelength of 480 nm (Figure S6a, Supporting Information). The concentration was then determined based on the absorption value and a calibration curve (Figure S6b, Supporting Information).

### Droplet Microfluidics

The microfluidic devices were fabricated on a silicon wafer (N100, University, USA) using maskless lithography (SF‐100 Xcel, Intelligent Micro Patterning, LLC, UK). SU‐8 photoresist (2025, MicroChem, USA) was used. The wafer was treated with trichloro(1H,1H,2H,2H‐perfluorooctyl)silane to form a uniform perfluorosilane coating, which increases the hydrophobicity of the surface. Next, the PDMS prepolymer base (Sylgard 184, Dow Corning, USA) was mixed with 10 wt.% of its curing agent using a conditioning mixer (AR‐100, THINKY, Japan, JP). The mixture was poured onto the silicon mold, and the wafer with PDMS was placed in a vacuum chamber for 30 min to remove air bubbles, followed by complete curing at 65 °C for 4 h. After curing, the PDMS channel was peeled off from the wafer and bonded to a glass slide (ISOLAB, Germany, DE) using oxygen plasma treatment (PDC‐002, Harrick, USA). The microfluidic devices are subject to heating at 90 °C for 2 h. Finally, the channel was treated with Aquapel (PPG, USA) to further increase the hydrophobicity of microfluidic channels, ensuring stable droplet generation.

Three syringe pumps (neMESYS, CETONI, DE) were employed to control the flow rates of inner and middle aqueous phases as well as the outer oil phase, respectively. The flow rates of inner and middle phases were set at a constant 200 µL/h, while the flow rate of the outer oil phase was adjusted between 200 and 1000 µL/h to control microdroplet sizes. The inner and middle aqueous phases converge at the first junctions, flowing side by side before being pinched off into microdroplets by the outer oil phase. All generated microdroplets were stabilized by fluorosurfactants (1 wt.%, Ran biotechnologies, Inc.) and collected in microcentrifuge tubes for subsequent microscopic imaging and analysis.

### Statistical Analysis

No statistical method was used to predetermine sample size. No data was excluded from the analysis. Each result presented in work was obtained after at least three independent experiments.

## Conflict of Interest

Ho Cheung Shum is a scientific advisor of EN Technology Limited, MicroDiagnostics Limited, PharmaEase Tech Limited, Upgrade Biopolymers Limited and Multera Limited, in which he owns some equity, and is a founding director and co‐director of the research center, Advanced Biomedical Instrumentation Centre Limited. The works in this paper are, however, not directly related to the works of these entities, as far as we know. The authors declare no other competing interests.

## Supporting information



Supporting Information

## Data Availability

The data that support the findings of this study are available from the corresponding author upon reasonable request.
