# Supplementary material for: Vortex‐Generated Microdroplets Enable Simple, Rapid, and Low‐Volume Measurement of Condensate Volume and Concentration
Source: Adv Sci (Weinh). 2025 Aug 21;12(42):e11197. doi: 10.1002/advs.202511197 (PMC12622474; doi:10.1002/advs.202511197)
Supplement: Supplementary file 1 — Supporting Information [file ADVS-12-e11197-s001.pdf]

Supplementary information for

Vortex-generated Microdroplets Enable Simple, Rapid, and Low-volume Measurement of Condensate Volume and Concentration

Feipeng Chen[1,2]*, Ho Cheung Shum[1,2,3]*

[1]Department of Mechanical Engineering, The University of Hong Kong, Pokfulam Road, Hong Kong (SAR) 999077, China

[2]Advanced Biomedical Instrumentation Centre, Hong Kong Science Park, Shatin, New Territories, Hong Kong (SAR) 999077, China

[3]Department of Biomedical Engineering & Department of Chemistry, City University of Hong Kong, Kowloon, Hong Kong, China.

*Corresponding author. Email: fpchen@connect.hku.hk (F.C.); ashum@cityu.edu.hk (H.C.S.)

This PDF file includes:

Supplementary Fig. 1 to Fig. 6

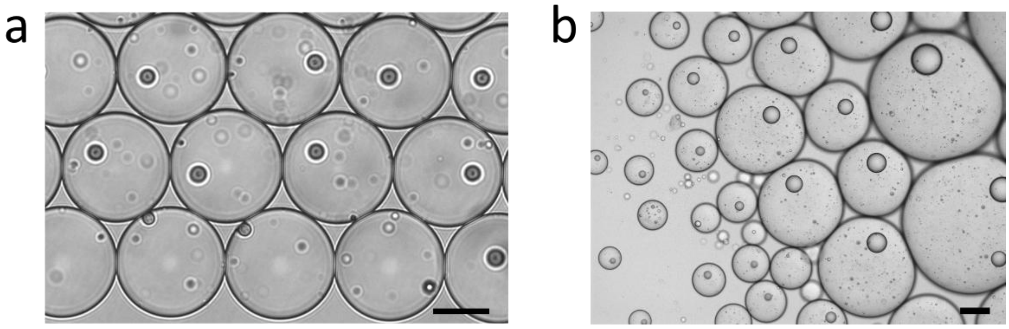

Supplementary Fig. 1. Optical images showing the presence of multiple small condensates dispersed within microdroplets after generation by (a) droplet microfluidics and (b) vortex-assisted emulsification. Scale bars are 50 μm.

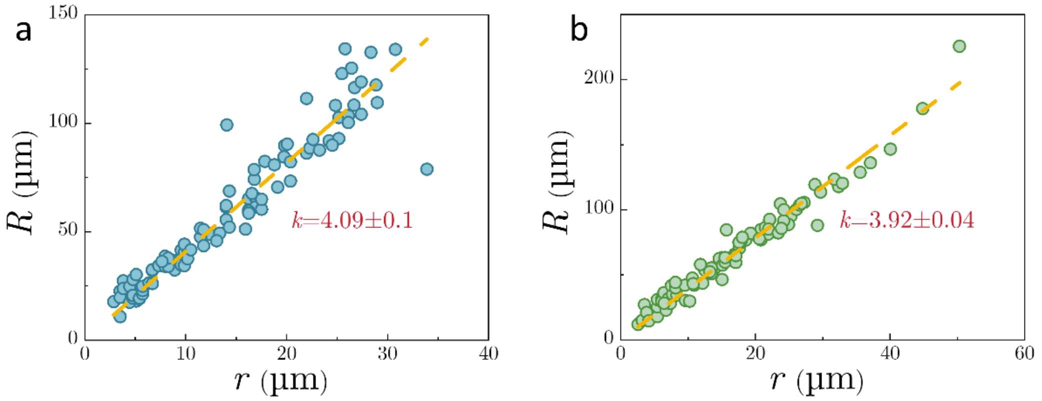

Supplementary Fig. 2. Plots of microdroplet radius (R) as a function of condensate radius (r) for samples prepared by vortex-assisted emulsification at different water/oil volume ratios: (a) 10/190 and 10/40, respectively.

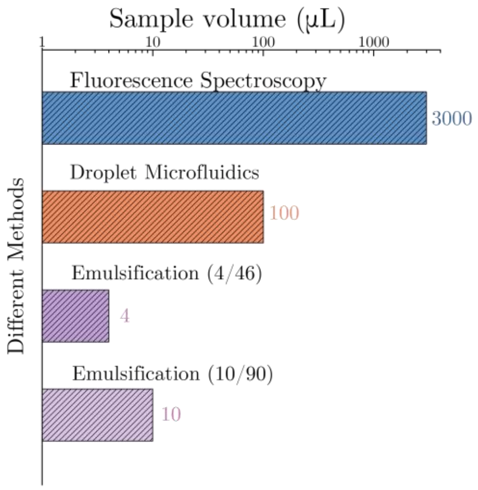

Supplementary Fig. 3. Comparison of sample volume required for measuring condensate concentration by different methods.

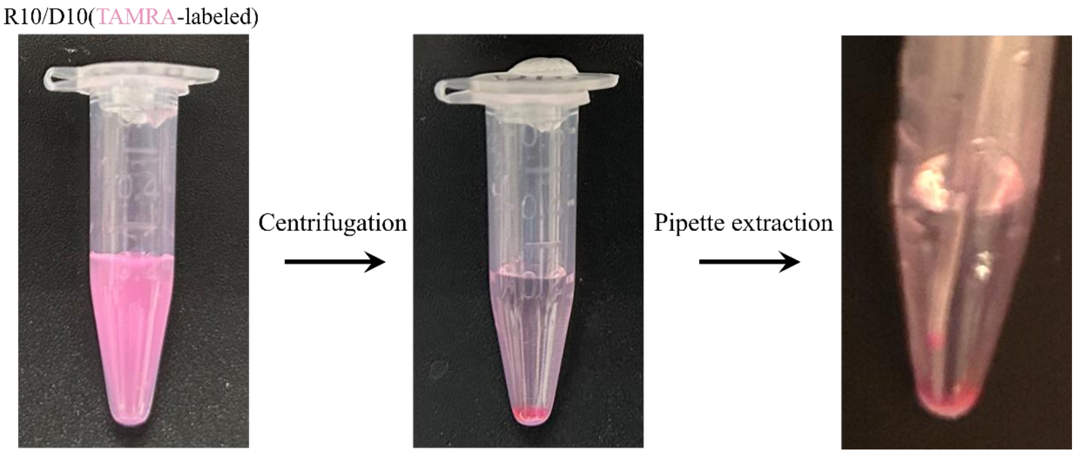

Supplementary Fig. 4. Photographs of R10/D10 condensates formed within a microcentrifuge tube. Here, D10 is labeled with the TAMRA fluorophore, showing a pink color. After centrifugation, the R10/D10 condensates adhere to the bottom of the tube and become difficult to extract them with a pipette in a precise volume.

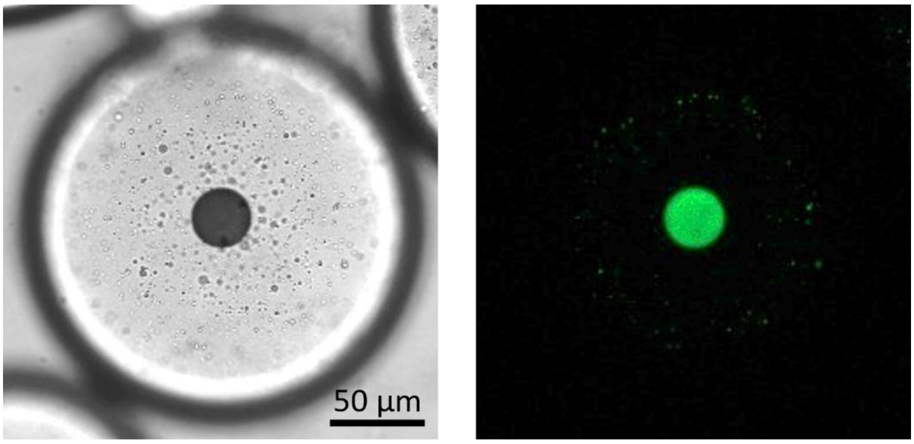

Supplementary Fig. 5. Optical and fluorescence images showing the stable R10/ATP condensates after two days. These condensates are prepared at a stoichiometric ratio of ATP to R10 at 3.2.

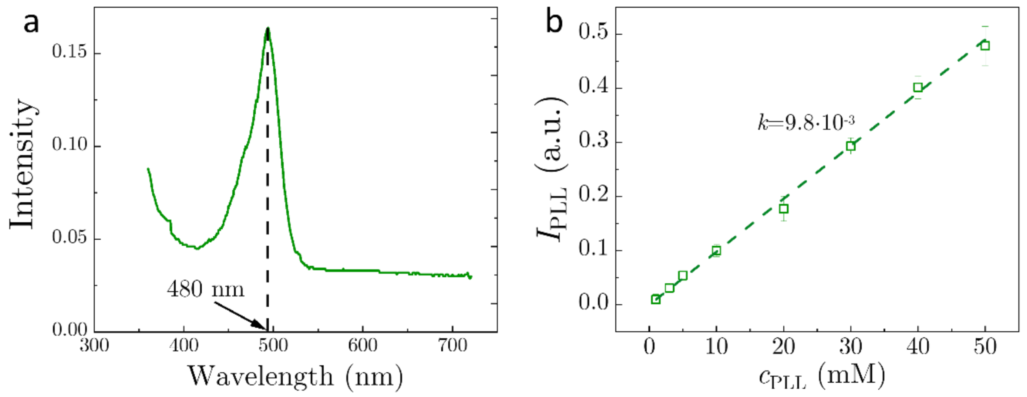

Supplementary Fig. 6. (a) Absorption spectrum of FITC-PLL at different wavelengths. A characteristic peak is identified at 480 nm. (b) A calibration curve of absorption intensity at the wavelength of 480 nm as a function of the concentration of FITC-PLL.
